# Hospital Notes

**Published:** 1893-06

**Authors:** 


### Hospital Notes.

The following changes have been made at the various Buffalo hospitals :

Fitch Hospital: Dr. J. J. Cullinane has been appointed to succeed Dr. Bradbury as senior house surgeon ; Mr. E. L. Puffer has been made first assistant.

Emergency Hospital: Dr. Fred. M. Boyle will take Dr. Culbert's position as resident physician. Dr. Frank J. Carr has become first assistant, and Dr. J. H. Walsh, second assistant.

At the Buffalo Hospital of the Sisters of Charity, Dr. Daniel J. White officiates as house physician, with Dr. Harry Newton as first assistant, and Dr. Patrick J. Hourigan, second assistant.

At the Buffalo General Hospital, Dr. Albert Woehnert and Dr. Frederick Mann succeed Drs. Woodruff and Hetzel as house physicians.

